# A tandem regression-outlier analysis of a ligand cellular system for key structural modifications around ligand binding

**DOI:** 10.1186/1758-2946-5-21

**Published:** 2013-04-30

**Authors:** Ying-Ting Lin

**Affiliations:** 1 Department of Biotechnology, College of Life Sciences, Kaohsiung Medical University, 100, Shih-Chuan 1st Road, San Ming District, 807, Kaohsiung City, Taiwan

## Abstract

**Background:**

A tandem technique of hard equipment is often used for the chemical analysis of a single cell to first isolate and then detect the wanted identities. The first part is the separation of wanted chemicals from the bulk of a cell; the second part is the actual detection of the important identities. To identify the key structural modifications around ligand binding, the present study aims to develop a counterpart of tandem technique for cheminformatics. A statistical regression and its outliers act as a computational technique for separation.

**Results:**

A PPARγ (peroxisome proliferator-activated receptor gamma) agonist cellular system was subjected to such an investigation. Results show that this tandem regression-outlier analysis, or the prioritization of the context equations tagged with features of the outliers, is an effective regression technique of cheminformatics to detect key structural modifications, as well as their tendency of impact to ligand binding.

**Conclusions:**

The key structural modifications around ligand binding are effectively extracted or characterized out of cellular reactions. This is because molecular binding is the paramount factor in such ligand cellular system and key structural modifications around ligand binding are expected to create outliers. Therefore, such outliers can be captured by this tandem regression-outlier analysis.

## Background

In any chemical analysis of a single cell, the first step is the separation of wanted chemicals from the bulk of a cell. This is due to the fact that a cellular system has a complex, heterogeneous composition. Various methods [1] using hard equipment have been developed for such uses. After a single cell is separated from the other cells, the wanted component can be further isolated and then detected through what is called a tandem technique [1]. The first part of a tandem technique, as mentioned above, is the separation of wanted chemicals from the bulk of a cell; the second part is the detection of the components. By mimicking such a tandem technique, a computational counterpart was developed herein; a statistical regression and its outliers (influential observations [2]) act as a computational technique for separation, which can cause the important identities (i.e. the factors causing outliers) to be isolated from the bulk of a cellular system. As a pioneer investigation, one molecular descriptor and one class of descriptors will be prepared: the descriptor resembles the filter in the tandem equipment; the class resembles the detector.

In a ligand-dependent receptor-mediated cellular system (or ligand cellular system), key structural modifications surrounding ligand binding are expected to cause outliers. For example, hydrogen bond formation, or deformation, can cause drastic alterations in cellular reaction. These singular situations are, at times, the reasons for statistical breakdown points in many analyses that are otherwise correct (i.e. resulting in the outliers of a statistical regression [3,4]). At the same time, such outliers can have the most prominent and often most informative features of the target-specific activity landscapes [5]. Therefore, the concept that after this tandem regression-outlier analysis, the features of these resulting outliers can correspond to important structural modifications around molecular binding in such a ligand cellular system, if correct, would be very useful.

For this first tandem part, we sought the most representative descriptor for the bulk system of a cell. We found Jurs_RNCG [6], after observing more than 521×17 data sets of PPARγ (peroxisome proliferator-activated receptor gamma) agonists [7-21]. The methods and results are depicted in the first body of materials, methods, and results. To connect this to the second tandem part, the descriptor that is sought in the first part has functionality, which yields outlier residues for the second part.

Acting as an assay for detection in the second tandem part, for which the electrotopological state (ES) class of descriptors [22-26] is used. All possible structural modifications in a given collected analog set are pre-assigned by ES descriptors. The ES descriptors involve atom types in various electro-topological states. For example, in ES terminology, an ES_Count_ssO of a molecular structure is the count of “ssO” linkages, and here the “ssO” represents a bonding oxygen atom (O) linked via two single bonds (ss). A structural modification is considered a fundamental element (an action) for the reaction of such a ligand cellular system. In actuality, any structural modification of such an analog set can be expressed by the change of an associated ES descriptor. The details and results of the second part are also depicted in the second body of materials, methods and results.

This tandem regression-outlier technique is, therefore, in mathematical terms, carried out so as to prioritize the context equations tagged with features of these outliers. We want to know if the top-ranked structural modifications correspond to the key interactions around molecular binding as we expected them to. This expectation was based on the fact that: I: this singular situation causes outliers in a regression. II: molecular binding is the paramount factor in such a ligand cellular system, and III, key structural modifications around ligand binding are expected to create singular situations, i.e. cause outliers in a statistical regression.

In the end, after this tandem regression-outlier analysis for the PPARγ agonist cellular system, a top ranked ES symbol can faithfully correspond to key interactions around molecular binding with the correct order of potency. The outcome of such an analysis confirmed the two main underlying and mutually-dependent speculations; one being that, in the second tandem part, the top ranked ES symbols reflect the key interactions around ligand binding, and the other that, in the first tandem part, the designation of Jurs_RNCG (relative negative charge) can effectively remove the general effects of such a ligand cellular system.

## Methods

### The first tandem filter: in order to seek the most representative descriptor for a ligand cellular system

In mathematics, the dependent variable Y is the ligand-dependent receptor-mediated cellular reaction. The context equation of the descriptor selection is given as follows:

(1) $$Y=\beta_{0}+\beta_{\mathrm{ch}}X_{\text{ch}}$$

where Y is the dependent variable that stands for the ligand-dependent receptor-mediated cellular reaction, X_ch_ is the descriptor to be chosen, and β_0_ and β_ch_ are the regression coefficients after the least squares fit. Once the ligand-dependent receptor-mediated cellular data of a given set of analogs are available, the r^2^ correlation fit can be obtained for each descriptor. Here, 521 descriptors of eminent classes are used. All descriptors in the working equation with correlation fits are prioritized by correlation coefficient. All calculations of descriptors were performed using the Discovery Studio 2.1 QSAR module [27]. The regression fits were conducted for each descriptor in the context equation and Pearson’s coefficients were performed using R 2.11.0 [28].

### The second tandem detector: to prioritize the context equations tagged with all possible features of the outliers

Following the designation of the Jurs_RNCG descriptor, a three-variable equation is used for the prioritization of all the ES descriptors. The context equation using Jurs_RNCG, tagged with all possible features of the outliers, is given as follows:

(2) $$Y=\beta_{0}+\beta_{\text{Jurs}\_\text{RNCG}}\text{Jurs}\_\text{RNCG}+\beta_{\text{ES}}\text{ES}$$

where Y is the dependent variable standing for the ligand-dependent receptor-mediated cellular reaction, Jurs_RNCG is the calculated Jurs descriptor, ES are all the possible ES descriptors; and all βs are the estimated regression coefficients after the least squares fit. The context equations tagged with all possible ES descriptors are prioritized by correlation coefficient. 12 top-ranked ES descriptors monitored in the table indicate 12 important structural modifications in a given analog set.

## Materials

### Three data sets of analogs with two cores

To demonstrate the ability of this tandem regression-outlier analysis to remove all interference from any general effects in a ligand cellular system, three data sets of the ligand-dependent receptor-mediated data are used here. The first data set is a collection of 46 PPARγ agonists with the thiazolidinedione (TZD) core. The second data set is composed of 178 PPARγ agonists with a carboxylic acid core. The third data set is a merger of the first and second data set (i.e., 224 PPARγ agonists mixed with both TZD and carboxylic acid cores). The two main cores of PPARγ agonists and their merger are adopted, so as to observe the variations of top-ranked structural modifications. All EC_50_ (50% efficacy concentration) data were extracted from the literature [7-21]. The cellular reaction is the measurement of the activation of PPARγ within the construct of the cellular transactivation assays. Indeterminate and uncertain EC_50_ values were excluded. A negative logarithm of the EC_50_ values of PPARγ agonists was then taken. The original publication of all agonists and the activity quantities are listed in Additional file 1: Tables S1 and S2. All images of molecular structures were created by using Pybel [29,30]. All molecular structures were energetically geometry-optimized using molecular mechanics and MMF97 calculations, which were implemented using the ChemBio3D software of the ChemBioOffice package [31].

## Results

### Jurs_RNCG as most representative descriptor

In the mathematical formulation in the first tandem filter, we used a large number data set, or 178 collected carboxylic acid PPARγ agonists, as a base to seek the most representative descriptor. More details about PPARγ agonists are available in Material Section. To check the size dependency, for each data size from 10 to 170, a total of 521 × 17 data sets were taken for the selection of the descriptor just for the enough randomness. Sample agonists of each size were picked out by prioritizing 521 molecular properties. The dominant descriptor and the frequency of the Jurs type descriptor for each size, including Jurs_RNCG [6], is listed in Table 1. For example, in the data size of 170 agonists, the dominant descriptors of 369 data sets among a total of 521 sets are all Jurs_RNCG. We can clearly see that when the data size increases, the Jurs_RNCG is more frequently dominant or near dominant (rank > 4). In Jurs terminology, RNCG means a relative negative charge [6].

**Table 1:** Dominant descriptors for each data size and the frequency of Jurs_RNCG (Jurs type descriptors) throughout 521 data sets are summarized here

**Data size**	**Dominant descriptor (Near dominant)**	**Frequency of Jurs descriptors (Jurs_RNCG/Jurs type /521 data sets)**
10	Molecular_PolarSASA	4/41/521
20	Molecular_FractionalPolarSASA	5/25/521
30	ES_Count_dO^b^	4/14/521
40	Num_RingBonds	10/14/521
50	SC_3_P^a^	9/16/521
60	SC_3_P^a^	9/12/521
70	IC^a^	18/31/521
80	IC^a^	18/33/521
90	Jurs_RNCG	21/31/521
100	IC^a^ (Jurs_RNCG)	19/31/521
110	Jurs_RNCG	16/21/521
120	Num_AtomClasses (Jurs_RNCG)	21/23/521
130	IC^a^ (Jurs_RNCG)	28/29/521
140	IC^a^ (Jurs_RNCG)	39/306/521
150	Jurs_RNCG	39/39/521
160	IC^a^ (Jurs_RNCG)	53/53/521
170	Jurs_RNCG	369/369/521

^a^ The descriptors SC_3_P and IC are calculated based on graph theory.

^b^ The descriptor ES_Count_dO is a fractional descriptor (The definition of a fractional descriptor is in the discussion section).

Agonists of each data set are selected out of the 178 carboxylic acid PPARγ agonists. The top-ranked descriptor of each set was selected from 521 descriptors of eminent classes.

In a realistic physical-chemical representation, one would prefer the Jurs_RNCG to the IC (Information Content) [32] as the most representative descriptor for all general effects. This is because Jurs_RNCG was originally designed based on the charge-related nature. The descriptor IC, as an index of graph theory, deals with the topological aspect in nature. Therefore, the Jurs_RNCG descriptor here is thought to be the most representative single descriptor for all general effects in the PPARγ agonist cellular system. After this designation, to our surprise, the Jurs_RNCG is further shown to be a linear combination of three important descriptors: LogD (partition coefficient), PSA (polar surface area), and shape-like descriptors in a subsequent work [33]. These three descriptors happen to be the three most important factors of investigation in medicinal chemistry over the past 50 years [34].

### Top-ranked ES descriptors as important structural modifications around ligand binding

Table 2 lists the 12 top-ranked ES descriptors for the 46 TZD PPARγ agonists after the conduct of prioritization in the second tandem detector. Table 3 lists the 12 top-ranked ES descriptors for the 178 carboxylic acid PPARγ agonists. Table 4, which is the merger of the two former sets, lists the 12 top-ranked ES descriptors for the 224 PPARγ agonists. The striking feature in all three tables is that the ES symbol, ssO, ranks in the first position. ‘s’ indicates a single bond. ‘ss’ indicates two single bonds linked in structure. This feature reflects a key interaction between potent agonist and PPARγ receptors, which shows concurrence with the inference of the X-ray crystallography depicted in the next section.

**Table 2:** The top-ranked ES descriptors of 46 TZD PPARγ agonists

**ES descriptors**	**Rank**	**Sign of β**_**ES**_
Count_ssO	1	-
Sum_ssO	2	-
Sum_sssN	3	+
Count_sssN	4	+
Count_ssCH2	5	+
Sum_aaO	6	+
Count_aaO	7	+
Count_sCH3	8	-
Sum_aaN	9	+
Count_aaN	10	+
Count_dsCH	11	-
Sum_dsCH	12	-

**Table 3:** The top-ranked ES descriptors of 178 carboxylic acid PPARγ agonists

**ES descriptors**	**Rank**	**Sign of β**_***ES***_
Sum_ssO	1	-
Count_ssO	2	-
Sum_aaO	3	+
Count_aaO	4	+
Count_aaaC	5	+
Sum_ssCH2	6	-
Count_aaaC	7	+
Count_ssCH2	8	-
Count_aaNH	9	+
Sum_aaNH	10	+
Count_ssssC	11	-
Sum_sssN	12	+

**Table 4:** The top-ranked ES descriptors of 224 PPARγ agonists with both TZD and carboxylic acid cores

**ES descriptors**	**Rank**	**Sign of β**_***ES***_
Count_ssO	1	-
Sum_ssO	2	-
Sum_aaO	3	+
Count_aaO	4	+
Sum_aaaC	5	+
Count_aaaC	6	+
Count_sssN	7	+
Sum_sssN	8	+
Count_aaNH	9	+
Sum_aaNH	10	+
Sum_aaN	11	+
Count_aaN	12	+

**Figure 1:**
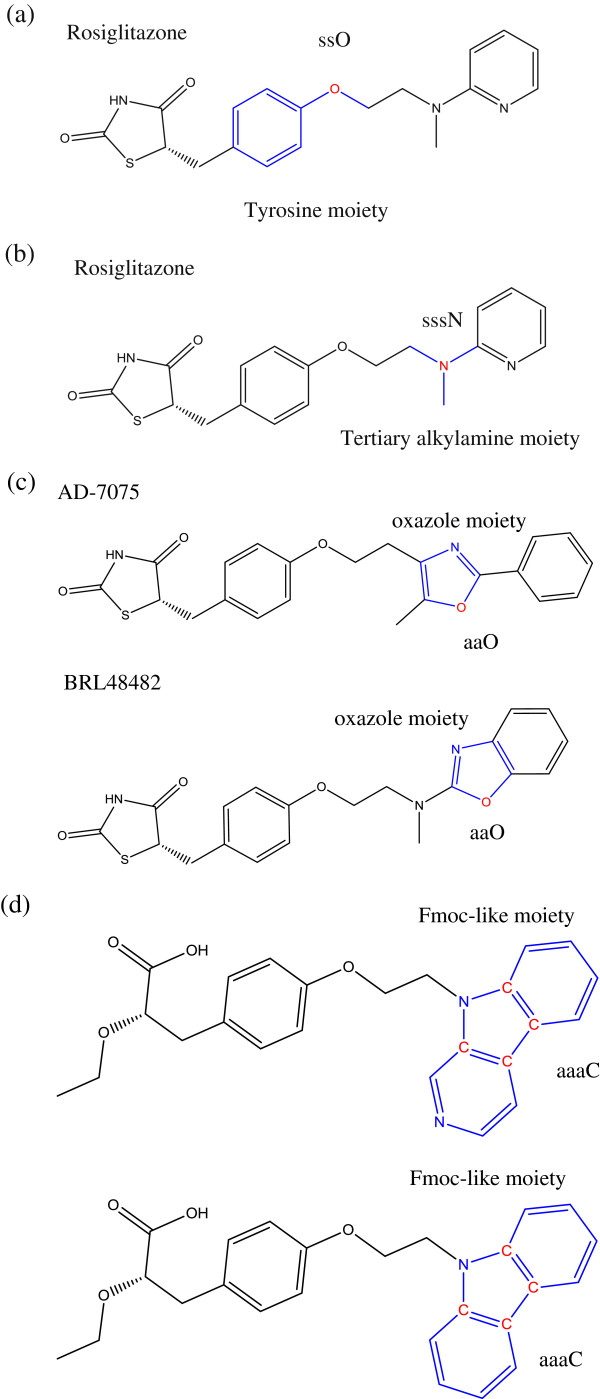
**Molecular structures of: (a) Rosiglitzaone with tyrosine moiety (ssO), (b) Rosiglitzaone with tertiary alkylamine moiety (sssN), (c) AD-7075 and BRL48482 with oxazole moiety (aaO), and (d) Two carboxylic acid agonists with Fmoc-like moiety (aaaC).** All moiety structures are colored in blue and the atom of the ES symbol is colored in red.

To begin with, in Table 2, the ES_Count_ssO and ES_Sum_ssO rank in most top positions with a negative regression coefficient. Basically, this is a negative structural modification with respect to cellular reaction. But care should be taken here: this does not mean that the existence of ssO moiety in the structure is bad. On the contrary, the existence of single ssO moiety is extremely good. (We can read this from the most potent leads which all have tyrosine moiety.) When examining the original data, each collected TZD PPARγ agonist has at least one ssO moiety in the structure, here known as an oxygen atom in tyrosine moiety. The correct interpretation for the sign of such a symbol here therefore is that the introduction of more than one ssO moiety importantly decreases cellular reaction. Rosiglitazone, an established drug that is one of the collected TZD agonists, shows this corresponding feature with the ES symbol, ssO in Figure 1(a).

Next, the ES symbol sssN, indicates the introduction of a tertiary amine (sssN) moiety, which is a positive structural modification. ‘sss’ indicates three single bonds linked in structure. Nine agonists have 1 sssN moiety of the 46 collected agonists. The corresponding feature of the ES symbol, sssN, in the tertiary amine (sssN) moiety, is shown in Figure 1(b).

The next ES symbol in Table 2 is ssCH2. The positive regression coefficient indicates that the elongation of a ligand structure through the addition of carbon moiety (ssCH2) is a positive structural modification. This topological elongation of the agonist makes a large impact to the molecular binding. However, we notice that the ssCH2 symbol is negative in Table 3. And, when combining two data sets in Table 4, the topological structural modification ssCH2 falls out of the monitor table. Apparently, the suitable length of the TZD agonist is optimal for cellular binding.

The ES symbol following this in Table 2 is aaO. ‘aa’ indicates oxygen atom in an aromatic ring. Throughout the whole 46 TZD agonists there are only two agonists: AD-7075 and BRL48482, in Figure 1(c), that have the oxazole moiety. We notice that these two agonists serve as very good examples of “analog outliers”, which bear specific feature of outliers The other ES symbols, aaO, aaN and aaCH, also indicate this oxazole moiety. The 5-methyl-oxazole of AD-7075 has the additional symbols aasC and sCH3 whereas the benzo-oxazole of BRL48482 has the additional symbol aaaC. Taken together, the significance of oxazole moieties are faithfully pointed out by these symbols. The other monitored symbols, sCH3 and dsCH, indicate other, less important, structural modifications.

Lastly, in Table 3 and Table 4, we find a similar picture regarding the potency order of structural modifications. The top ES symbol, ssO, represents the most important structural modification, tyrosine moiety, performed on the middle part of PPARγ agonist. The rest of the top-ranked ES symbols represent important structural modifications done to the tail part. For example, a fmoc-like moiety (fluorenylmethyloxycarbonyl-like), other than oxazole moiety and tertiary amine, is another case which has an ES symbol of significance: aaaC. Again, serving as analog outliers, these two potent agonists [18] are shown in Figure 1(d).

There is one more observation: we can clearly see that no ES symbol regarding TZD moieties, dssC, dO, sssCH, ssNH, or ssS, appears in Table 2; and we can also see that no carboxylic acid symbols, such as dssC, dO, or sOH, appear in Table 3. ‘d’ indicates double bond. When combining the two data sets, no ES symbols regarding TZD or carboxylic acid appear in Table 4. There are two simple interpretations of this: 1, the lack of modification of the core part of analogs in a given set will naturally lead to no ES symbols monitored in the table, and 2, a core shift in the combined set without causing a large difference of reaction will not produce related ES symbols of significance. Thus, in conclusion, the TZD and carboxylic acid are known as the necessary parts of full PPARγ agonists without synthetic modifications. The necessity of two essential cores for full cellular activity can be immediately inferred when comparing the inactive compounds at initial synthesis.

### The top-ranked ES symbols point out key ligand binding interactions

**Figure 2:**
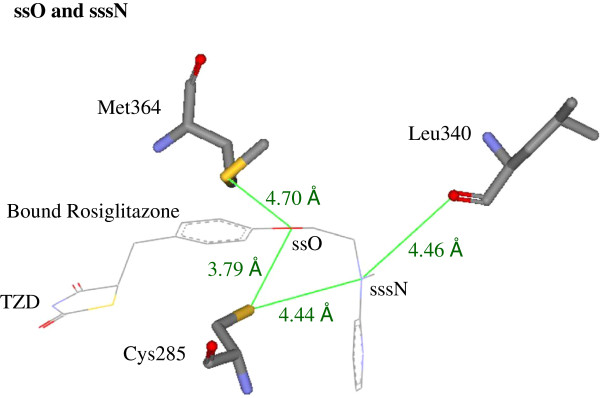
**Illustration of the top-ranked ES symbols, ssO and sssN.** Around the bound structure of rosiglitazone (PDB: 2PRG), the ES symbol ssO can detect the key interaction between rosiglitazone ether oxygen and the two sulfurs of Cys285 (3.79 Å) and Met364 (4.70 Å). Another important ES symbol, sssN, detects the key interaction between rosiglitazone trialkylamine nitrogen and Cys285 side-chain sulfur (4.44 Å), or Leu340 backbone oxygen (4.46 Å). Notice that all the measured distances are between relevant heavy atoms because hydrogen is transparent in the X-ray crystallography.

As mentioned in the introduction, we would like to see if the top-ranked ES descriptor finds its corresponding key interactions for molecular binding. In this section we examine real physical pictures. First, in Table 2, the top-ranked ES symbols are ssO and sssN. We can go to the crystallographic image of the rosiglitazone-PPARγ complex (PDB code: 2PRG) [35]. Figure 2 shows, around the bound structure of rosiglitazone, that the ES symbol ssO can detect the key interaction between rosiglitazone ether oxygen and two sulfurs of Cys285 (3.79 Å) and Met364 (4.70 Å). Another important ES symbol, sssN, detects the key interaction between rosiglitazone tertiary amine nitrogen and Cys285 side-chain sulfur (4.44 Å), or Leu340 backbone oxygen (4.46 Å). We noticed that all the measured distances are between relevant heavy atoms because hydrogen is transparent in X-ray crystallography.

**Figure 3:**
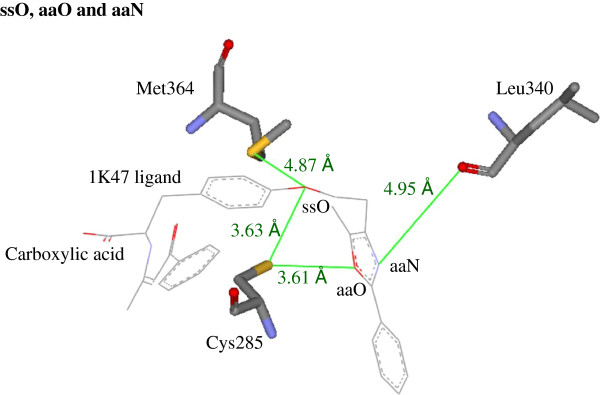
**Illustration of the top-ranked ES symbols ssO, aaO, and aaN.** Around a bound structure of 1K47 ligand (PDB: 1k47), the important ES symbol ssO can detect the key interaction between 1K74 ligand ether oxygen and Cys285 side-chain sulfur (3.63 Å) or Met364 sulfur (4.87 Å). Another key ES symbol, aaO, detects the key interactions between 1K74 ligand oxazole oxygen and Cys285 side-chain sulfur (3.61 Å). Here we also measured the distance (4.95 Å) between oxazole nitrogen (aaN) and Leu340 backbone oxygen.

Second, in Table 3, the top-ranked ES symbols are ssO and aaO. We then look at the crystallographic image of the 1K74 ligand-PPARγ complex (PDB code: 1K47) [36]. Figure 3 shows that, around the bound structure of the 1K47 ligand, a important ES symbol, ssO, can detect the key interaction between 1K74 ligand ether oxygen and Cys285 side-chain sulfur (3.63 Å) or Met364 sulfur (4.87 Å). Another important ES symbol, aaO, detects the key interactions between 1K74 ligand oxazole oxygen and Cys285 side-chain sulfur (3.61 Å). Here we also measured the distance (4.95 Å) between oxazole nitrogen (aaN) and Leu340 backbone oxygen. The symbol, aaN, as a important structural modification of the tail part, is indicated in Tables 2 and 4. More ES symbols regarding the tail part modifications of PPARγ agonists, as mentioned above, can also detect their corresponding key interactions in other crystallographic images of potent PPARγ agonists.

Taken together, these correspondences clearly point out that the top-ranked ES symbols are the key structural modifications surrounding molecular binding.

## Discussion

### Jurs_RNCG as a filter

The descriptor Jurs_RNCG acts as a filter. One might expect to see some outcomes if the single Jurs_RNCG descriptor is not included, i.e. there is no first filter of this tandem technique. Apparently, all general effects will contribute to the top-ranked ES descriptor. In Additional file 1: Table S3, for example, the top-ranked ES symbol, ssO, tyrosine moiety, of PPARγ agonists, falls outside the monitor table. In other words, we need a descriptor in the first regression that can effectively remove the general effect of a ligand cellular system. As mentioned above, one purpose for using Jurs_RNCG is to leave outliers for the second part.

Moreover, the potency orders and tendencies (signs of regression coefficients) of structural modifications coincide with our knowledge about the structural modifications of PPARγ agonists. So the top-ranked structural modifications can detect their corresponding key interactions surrounding molecular binding, as shown in the X-ray image of a potent agonist-PPARγ complex. The outcomes of such a regression-outlier analysis also tell us that the Jurs_RNCG is truly an adequate filter.

In addition, and exceeding our expectations, the Jurs_RNCG can be expressed in a linear combination of partition coefficients, polar surface area, and shape-like descriptors [33], which further reveals three essential factors for drug-cell interfaces in such a ligand cellular system [34].

### Three types of dependency in the top-ranked ES descriptors

For a closer inspection of the top-ranked ES descriptors, the ES symbols from Table 2 were intentionally combined in a single fitting equation. Three fitting equations are listed in Table 5. First, in Equation 1 of Table 5, the ES symbol Sum_ssO, has a positive regression coefficient opposite to the sign presented in Table 2. In Equation 2, the symbol Count_sssN, has a negative regression coefficient opposite to the sign provided in Table 2. At the same time, we noticed that the correlation (r) between Count_ssO and Sum_ssO in this 46 TZD data set is +0.98 and the correlation (r) between Count_sssN and Sum_sssN is +0.99; i.e., they have extremely significant positive correlations. In the interest of realistic representation, it is impossible that an identical important structural modification be represented by two highly positive-correlated descriptors indicating different tendencies. However, the fitting regression coefficient resulting in the different signs in these 2 equations is obvious. Therefore, the Count_ssO and Sum_ssO (or, the Count_sssN and Sum_sssN) have a dependency of description on identical structural modification.

**Table 5:** **The ES symbols monitored in the Table**2**are intentionally combined in a single fitting equation**

**#**	**Equation**
1	*Y* = 4.97 − 20.8*Jurs* _ *RNCG* − 2.81 *Count* _ *ssO* + 0.38 *Sum* _ *ssO* *
*2*	*Y* = 3.41 − 4.50 *Jurs_RNCG* − 3.09 *Count_ssO* + 0.47 *Sum_ssO* * + 0.95 *Sum_sssN* − 0.86 Count_ssN *
*3*	*Y* = 3.34 − 11.1 *Jurs_RNCG* − 0.33 *Count_ssO + 0.31Count_sssN* + 0.47 *Count_aaO* − 0.33 *Count_sCH*3 − 0.079 *Count_aaN* *

*The coefficient signs of these ES symbols labeled with star signs (*) contradict the ones presented in Table 2.

Three fitting equations with signs of regression coefficients are presented.

Second, by removing the modification description dependency, the Count and Sum values of the same ES symbol are not in the same equation. In Equation 3 of Table 5, the symbol Count_aaN has a negative regression coefficient compared to the sign listed in Table 2. It therefore contradicts the observation that the captured symbols, aaO, aaaC, sssN, aaNH, and aaN, represent positive structural modifications to the tail part of PPARγ agonists. Obviously, in this equation form 3, the symbols aaO and sssN represent the identical key interaction in the tail part of PPARγ agonists, and the simultaneous appearance of them for the same moiety turned the regression coefficient of the additional aaN into the opposite sign. Thus, one can say here that the ES symbols aaN, aaO and sssN have dependency of description on the identical moiety.

Third, throughout all of the 46 TZD PPARγ agonists, when examining the values of the symbol aaO and related structural moieties, we found that no structural moiety contains this aaO feature aside for oxazole. The moiety oxazole exists only in the two potent agonists AD-7057 and BRL48482 [10]. The value of ES_Count_aaO is 1 for these 2 agonists whereas the value is 0 for the rest of collected PPARγ agonists. The ES symbols of oxazole have aaO, aaN, and aaCH. Four agonists have the aaN structural moiety of these 46 collected agonists and, among those four, two compounds are AD-7057 and BRL48482. Moreover, all agonists have the aaCH structural moiety, but the symbol aaCH does not appear in Table 2. Clearly, the symbols aaO, aaN and aaCH have unequal dependencies of description in these data samples.

Especially, these dependencies of descriptor will actually cause serious consequence to all QSARs of four categories (classical, 3-dimensional, decisional and orthogonal) [37-39], their existence would make a model lose its interpretability. Put together, the three types of dependencies in the top-ranked ES symbols actually play a major role in the design of the context equation. That is, two ES symbols don’t appear simultaneously in a context equation. Obviously, if one forces two dependent ES descriptors to be combined in a single equation, the signs in the regression coefficients of key structural modifications may change, and thus fail to point out the real tendency of impact to ligand binding in an analog set. If one mixes two ES descriptors in a single equation acting as a detector, the one in this regression-outlier analysis will lose its ability to correctly detect the real tendencies of key structural modifications in the given analog sets.

## Conclusions

The innovative point of the present study is the fact that we used a statistical regression and its outlier as a computational technique for separation. This technique was used specifically in the ligand cellular system. As a counterpart to the hard equipment in the tandem technique, the prior molecular descriptor resembles a filter that removes the influence from the bulk of a cell and the latter class of descriptors is an array of detectors that can identify any important identities. In the case of the PPARγ agonist cellular system, the key structural modifications surrounding ligand binding were successfully detected and the tendencies of impact were examined. In the end, after the tandem regression-outlier analysis of this ligand cellular system, the results show that this prioritization of the context equations (filter) tagged with features of outliers (detector) is an effective computational tool for cheminformatics to detect possible features of outliers (key structural modifications), as well as their impact tendencies to ligand binding.

## Competing interests

The author declares that he has no competing interests.

## Supplementary Material

Additional file 1: Table S1 Lists the structures of the TZD PPARγ agonists with their activities. All images of molecular structures were created by using Pybel [29,30]. **Table S2** lists the structures of the Carboxylic (COOH) PPARγ agonists with their activities. All images of molecular structures were created by using Pybel [29,30]. **Table S3** lists the top-ranked ES descriptors of 178 carboxylic acid PPARγ agonists against cellular reactions, without the inclusion of Jurs_RNCG. Click here for file
