# Supplementary material for: A tandem regression-outlier analysis of a ligand cellular system for key structural modifications around ligand binding
Source: J Cheminform. 2013 Apr 30;5:21. doi: 10.1186/1758-2946-5-21 (PMC3648400; doi:10.1186/1758-2946-5-21)
Supplement: Additional file 1: Table S1 — Lists the structures of the TZD PPARγ agonists with their activities. All images of molecular structures were created by using Pybel [29,30]. Table S2 lists the structures of the Carboxylic (COOH) PPARγ agonists with their activities. All images of molecular structures were created by using Pybel [29,30]. Table S3 lists the top-ranked ES descriptors of 178 carboxylic acid PPARγ agonists against cellular reactions, without the inclusion of Jurs_RNCG. [file 1758-2946-5-21-S1.docx]

Additional Material

Note that in the two following tables, the abbreviation, *BMCL_03_1801_11*, stands for the compound, *Bio & Med Chem Letters, year 2003, page 1801, compound # 11*, BMC_03_4059_10 stands for the compound, *Bio & Med Chem, year 2003, page 4059, compound # 10*, and JMC_00_4_Pioglitazone stands for the compound, *Journal of Medicinal Chemistry, year 2000, page 4, compound ID Pioglitazone*.

**Table S1**. The TZD PPARγ agonists with their activities.

| TZD |  | Activity |
| --- | --- | --- |
| BMCL_03_1801_11 | 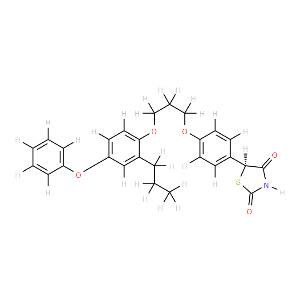 | 0.19 |
| BMCL_03_1801_12 | 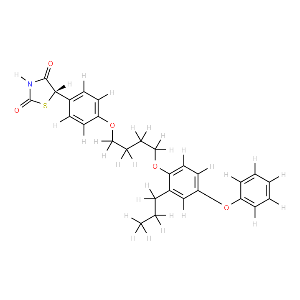 | -0.33 |
| BMCL_03_1801_15 | 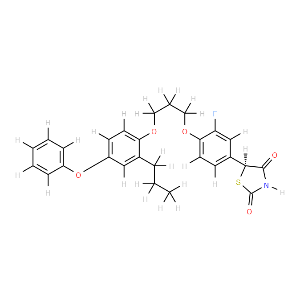 | 0.19 |
| BMCL_03_1801_16 | 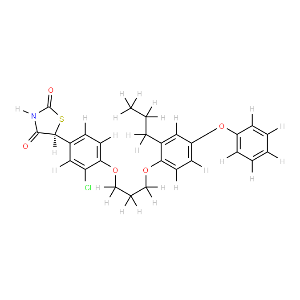 | -0.18 |
| BMCL_03_1801_17 | 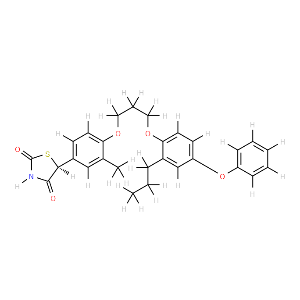 | 0.67 |
| BMCL_03_1801_18 | 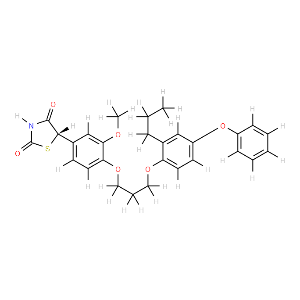 | 0.07 |
| BMCL_03_1801_19 | 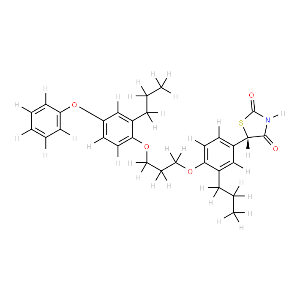 | 0.37 |
| BMCL_03_1801_22 | 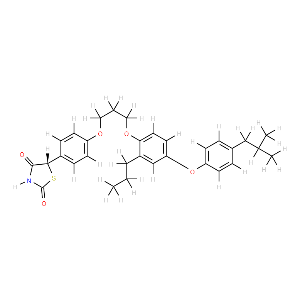 | 1.19 |
| BMCL_03_1801_24 | 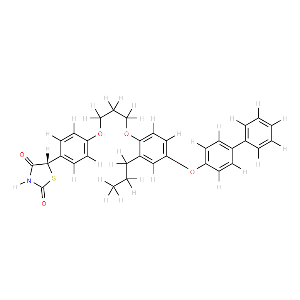 | 1.37 |
| BMCL_03_1801_25 | 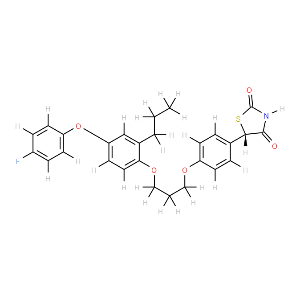 | 0.89 |
| BMCL_03_1801_26 | 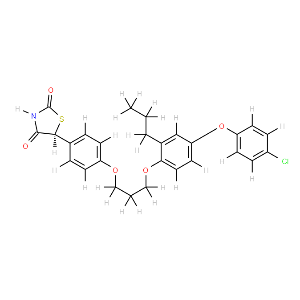 | 0.76 |
| BMCL_03_1801_27 | 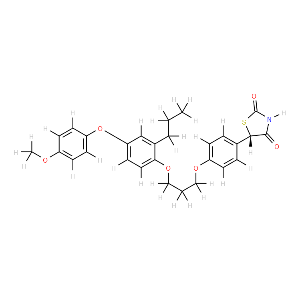 | 1.19 |
| BMCL_03_1801_28 | 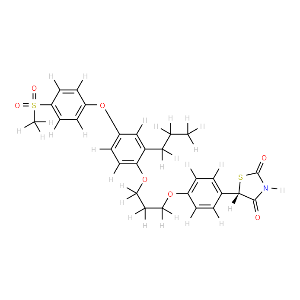 | 0.59 |
| BMCL_03_1801_30 | 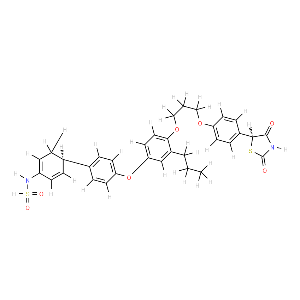 | -0.03 |
| BMCL_03_2795_11 | 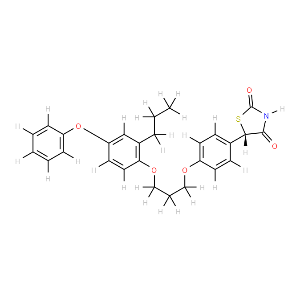 | 0.19 |
| BMCL_03_2795_12 | 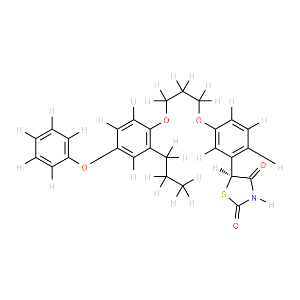 | 1.52 |
| BMCL_03_2795_15 | 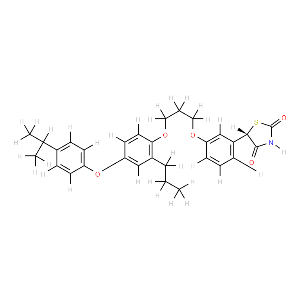 | 0.44 |
| BMCL_03_2795_18 | 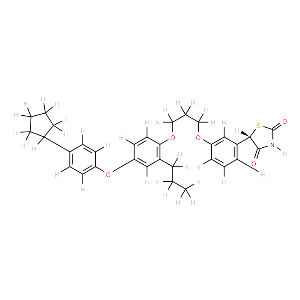 | 0.58 |
| BMCL_03_2795_19 | 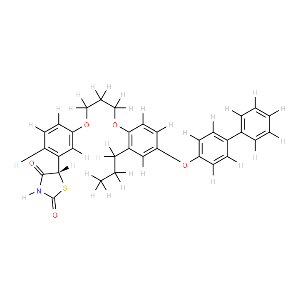 | 0.12 |
| BMCL_03_2795_20 | 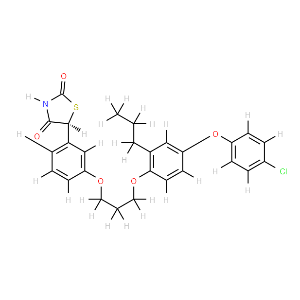 | 0.45 |
| BMCL_03_2795_21 | 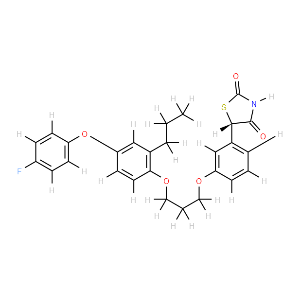 | 0.74 |
| BMCL_03_2795_22 | 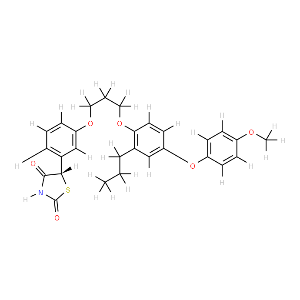 | 0.83 |
| BMCL_03_2795_23 | 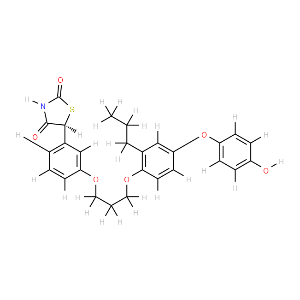 | 1.31 |
| BMCL_03_2795_24 | 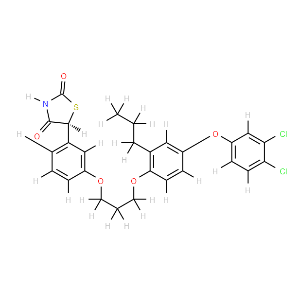 | 1.00 |
| BMCL_03_2795_25 | 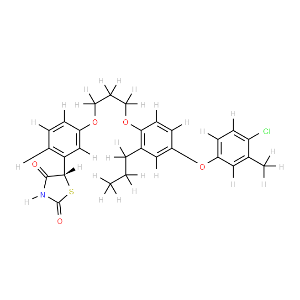 | 1.24 |
| BMCL_03_2795_26 | 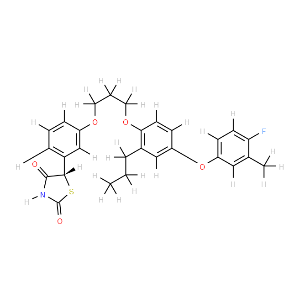 | 0.64 |
| BMC_03_4059_10 | 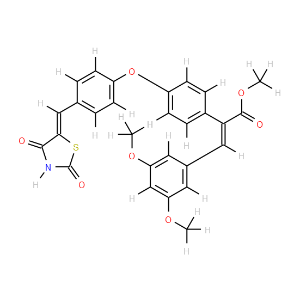 | -0.73 |
| BMC_03_4059_11 | 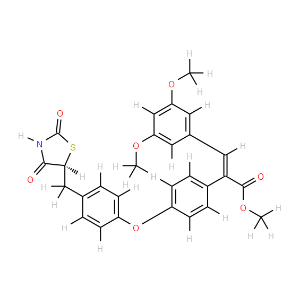 | -0.13 |
| BMC_03_4059_14 | 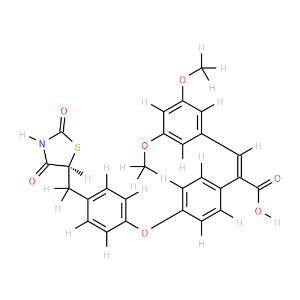 | -0.52 |
| BMC_03_4059_17 | 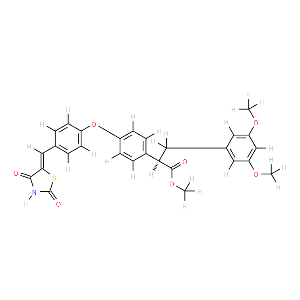 | -2.06 |
| BMC_03_4059_18 | 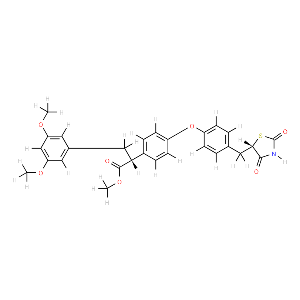 | -2.44 |
| BMC_03_4059_23 | 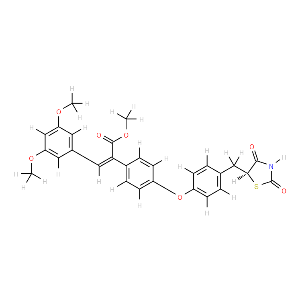 | -1.25 |
| JMC_00_527_KRP-297 | 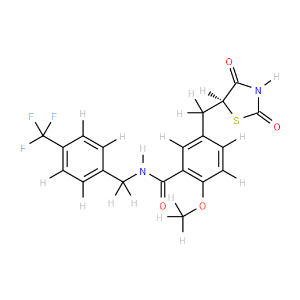 | 1.08 |
| JMC_00_527_Pioglitazone | 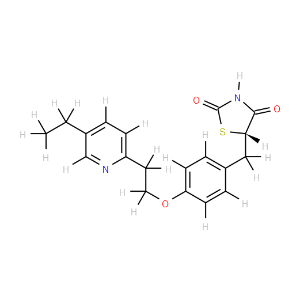 | 0.24 |
| JMC_00_527_Rosiglitazone | 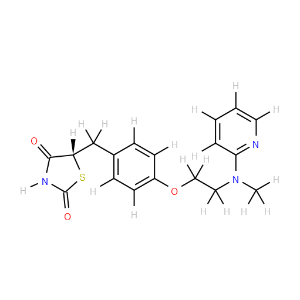 | 1.37 |
| JMC_00_527_Troglitazone | 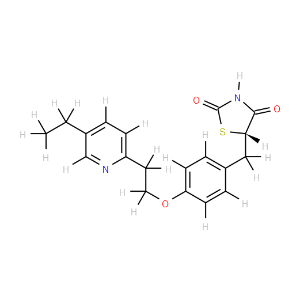 | 0.26 |
| JMC_06_4072_11 | 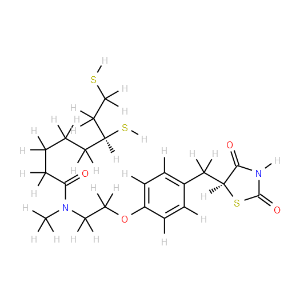 | 1.37 |
| JMC_06_4072_11a | 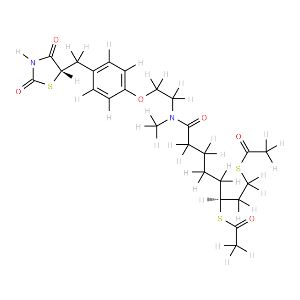 | 1.66 |
| JMC_06_4072_11b | 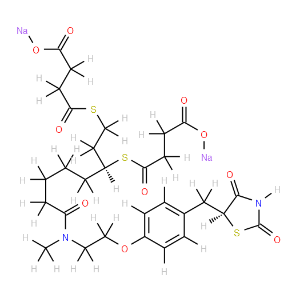 | 1.34 |
| JMC_06_4072_11c | 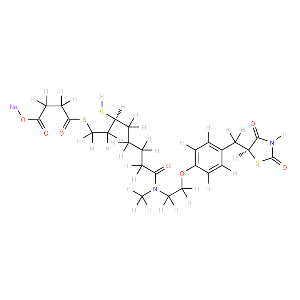 | 1.95 |
| JMC_06_4072_11d | 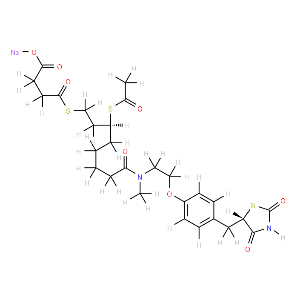 | 1.07 |
| JMC_06_4072_11e | 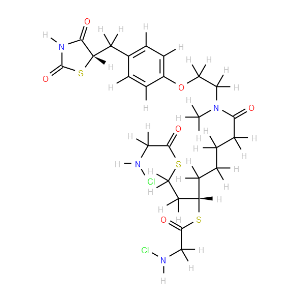 | 1.47 |
| JMC_06_4072_11f | 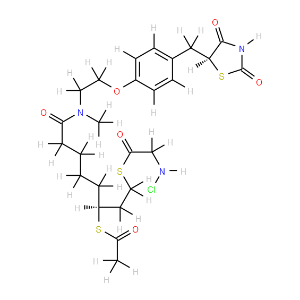 | 0.96 |
| JMC_98_5020_AD-7057 | 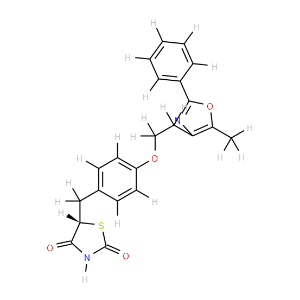 | 2.82 |
| JMC_98_5020_BRL48482 | 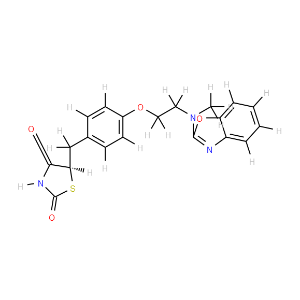 | 2.27 |
| JMC_98_5020_Ciglitazone | 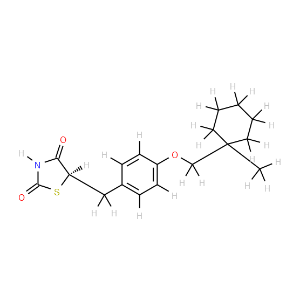 | -1.04 |

**Table S2.** The COOH PPARγ agonists with their activities.

| COOH |  | Activity |
| --- | --- | --- |
| BMCL_05_1497_001 | 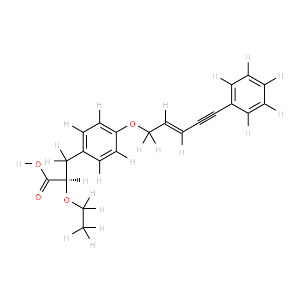 | 0.63 |
| BMCL_05_1497_002 | 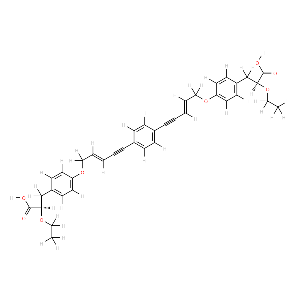 | 1.67 |
| BMCL_05_1497_003 | 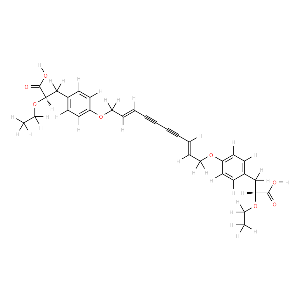 | 0.59 |
| BMCL_05_1497_004 | 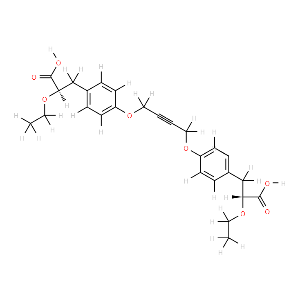 | -0.51 |
| BMCL_05_1497_005 | 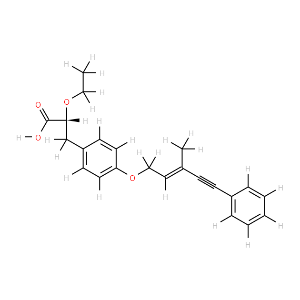 | 0.89 |
| BMCL_05_1497_006 | 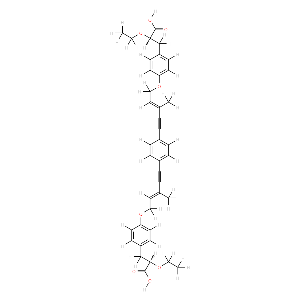 | 1.76 |
| BMCL_05_1497_007 | 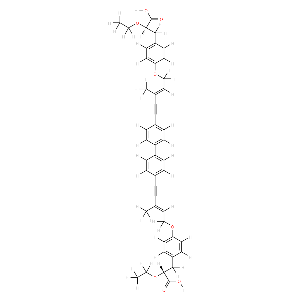 | 1.37 |
| BMCL_05_1497_008 | 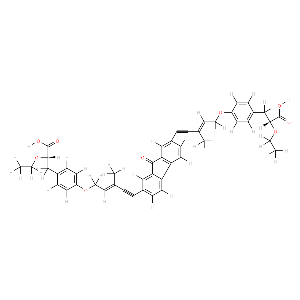 | 0.63 |
| BMCL_05_1497_009 | 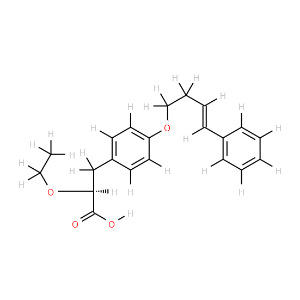 | 0.16 |
| BMCL_05_1497_011 | 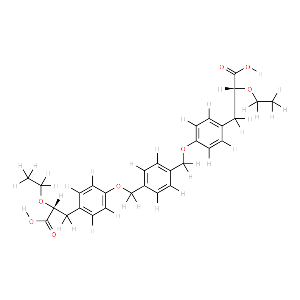 | -0.48 |
| BMCL_05_1497_012 | 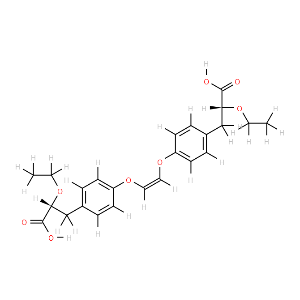 | -0.51 |
| BMCL_05_1497_013 | 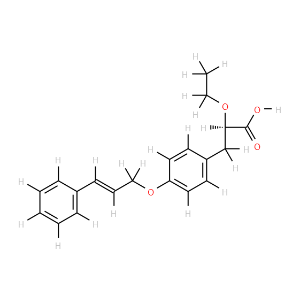 | -0.23 |
| BMCL_05_1497_014 | 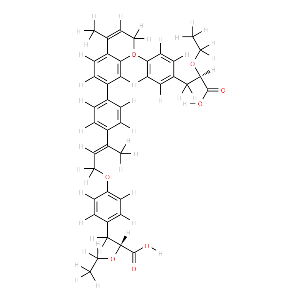 | 1.19 |
| BMCL_05_2437_001 | 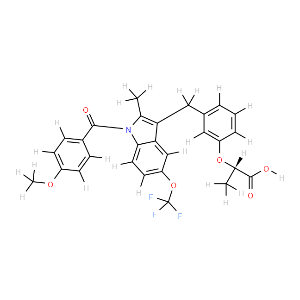 | 2.19 |
| BMCL_05_2437_004 | 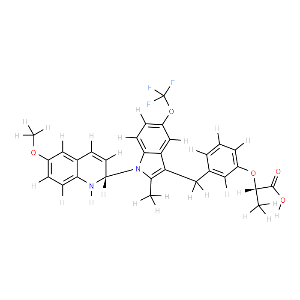 | 0.98 |
| BMCL_05_2437_005 | 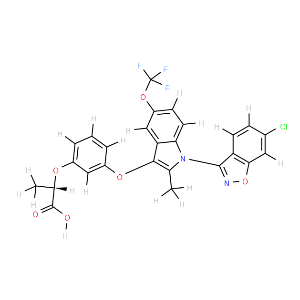 | 0.38 |
| BMCL_05_2437_007 | 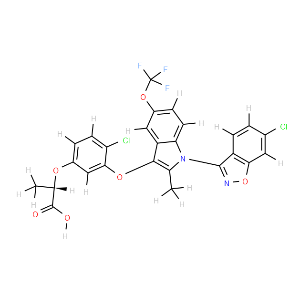 | 2.19 |
| BMCL_05_2437_008 | 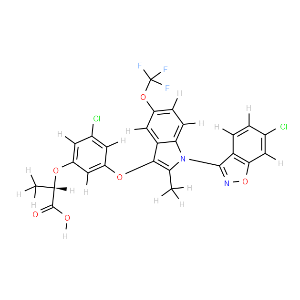 | 0.61 |
| BMCL_05_2437_009 | 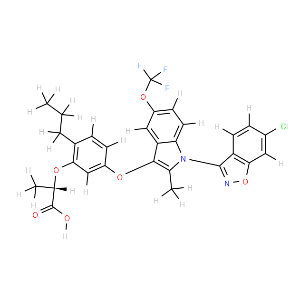 | 1.08 |
| BMCL_05_2437_010 | 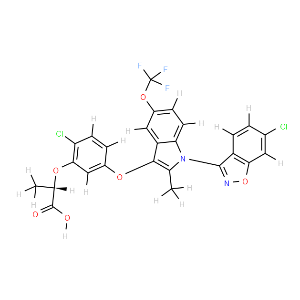 | 2.19 |
| BMCL_05_2437_012 | 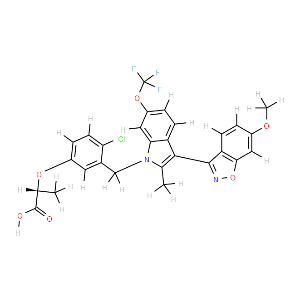 | 2.19 |
| BMCL_05_51_001a | 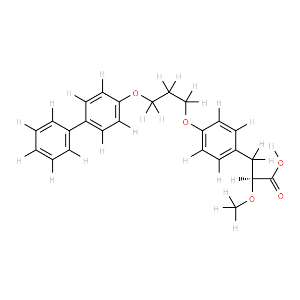 | 1.61 |
| BMCL_05_51_001b | 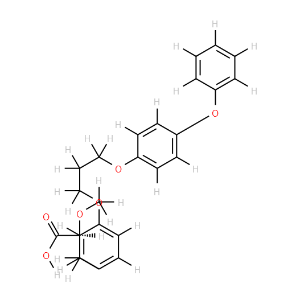 | 1.24 |
| BMCL_05_51_001c | 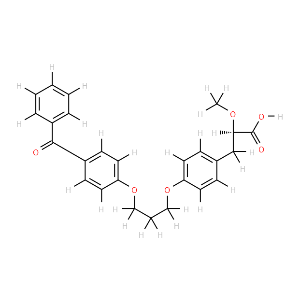 | 1.38 |
| BMCL_05_51_001d | 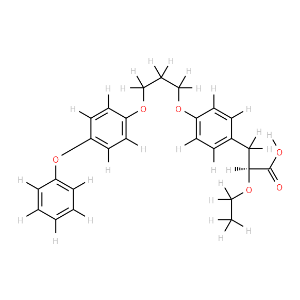 | 1.34 |
| BMCL_05_51_001e | 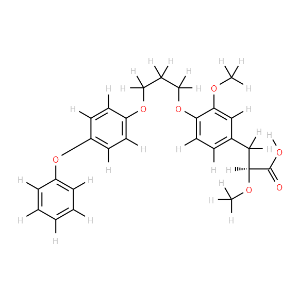 | 1.53 |
| BMCL_05_51_001f | 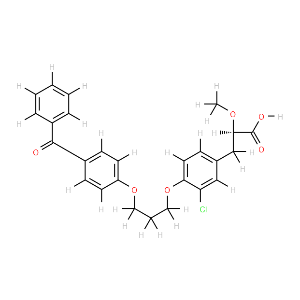 | 1.31 |
| BMCL_05_51_001g | 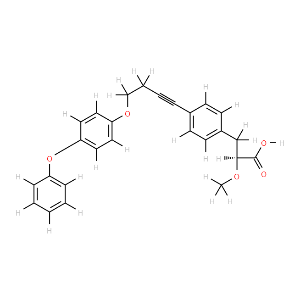 | 1.78 |
| BMCL_05_51_001h | 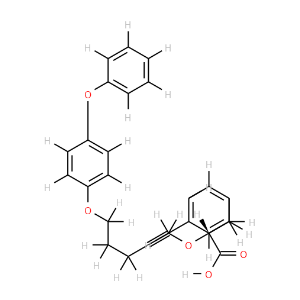 | 1.42 |
| BMC_06_866_006a | 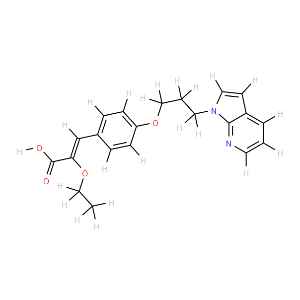 | 0.42 |
| BMC_06_866_006b | 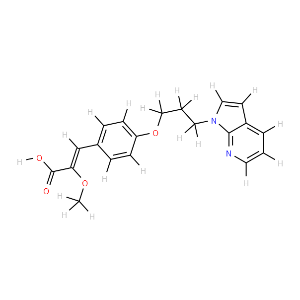 | 0.05 |
| BMC_06_866_006c | 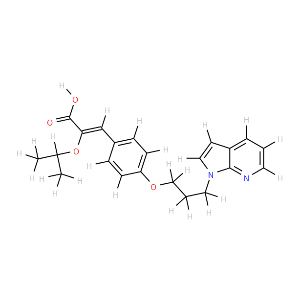 | 0.42 |
| BMC_06_866_006d | 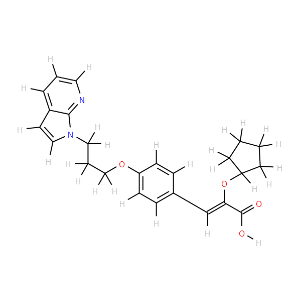 | 0.26 |
| BMC_06_866_006e | 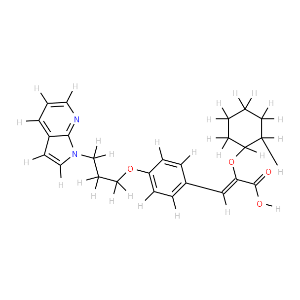 | 0.31 |
| BMC_06_866_006f | 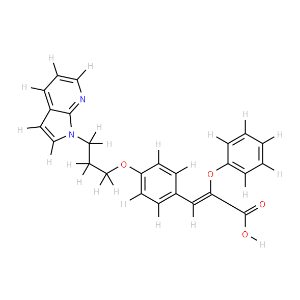 | 0.05 |
| BMC_06_866_006g | 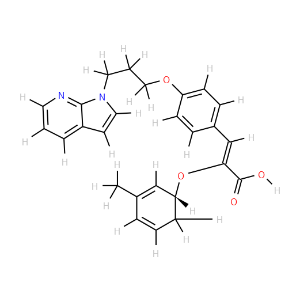 | 0.15 |
| BMC_06_866_006h | 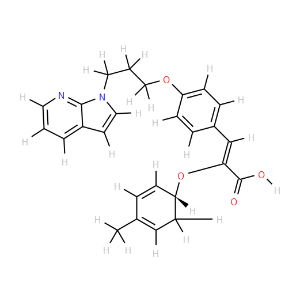 | 0.29 |
| BMC_06_866_006i | 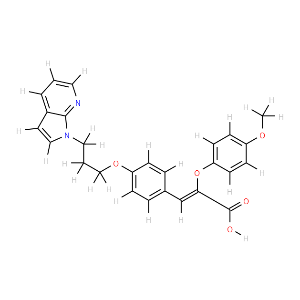 | 0.11 |
| BMC_06_866_006j | 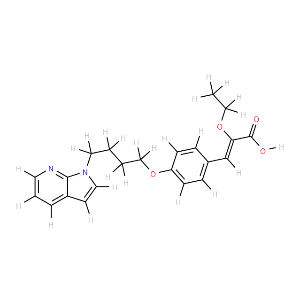 | -0.53 |
| BMC_06_866_006k | 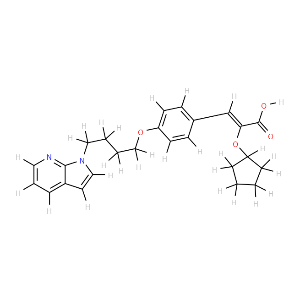 | -0.45 |
| BMC_06_866_006l | 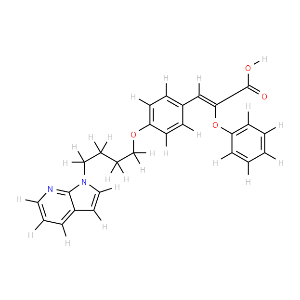 | -0.58 |
| BMC_06_866_007a | 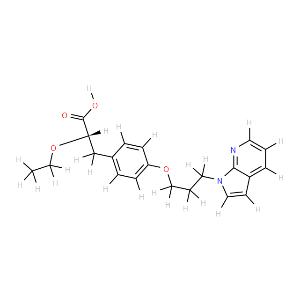 | 1.16 |
| BMC_06_866_007b | 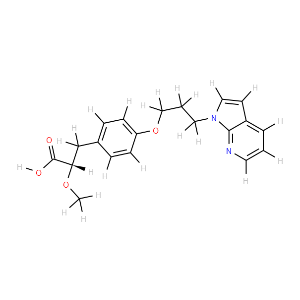 | 1.24 |
| BMC_06_866_007c | 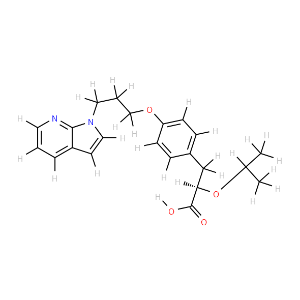 | 1.82 |
| BMC_06_866_007d | 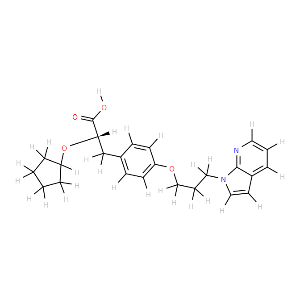 | 0.21 |
| BMC_06_866_007e | 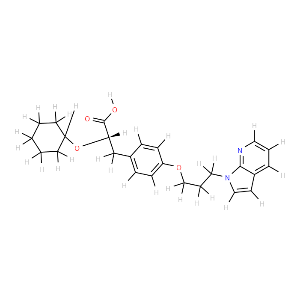 | -0.27 |
| BMC_06_866_007f | 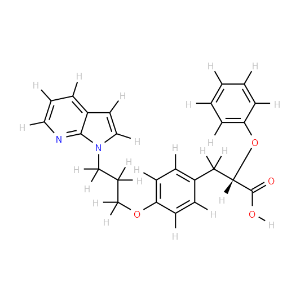 | -0.44 |
| BMC_06_866_007g | 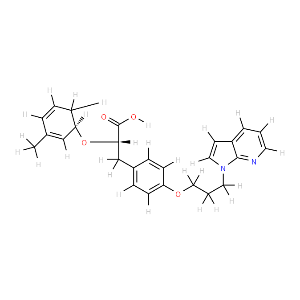 | -0.37 |
| BMC_06_866_007h | 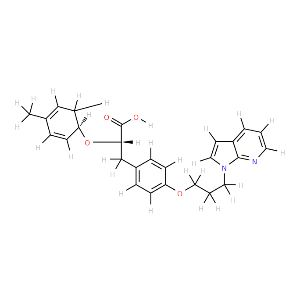 | -0.53 |
| BMC_06_866_007i | 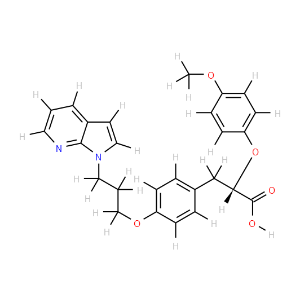 | -0.55 |
| BMC_06_866_007j | 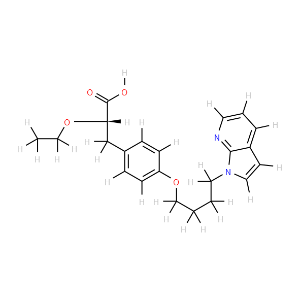 | 0.08 |
| BMC_06_866_007k | 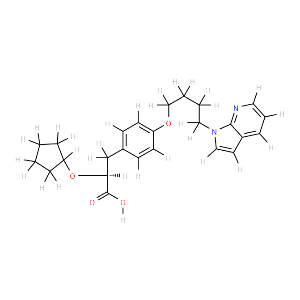 | -0.31 |
| BMC_06_866_007l | 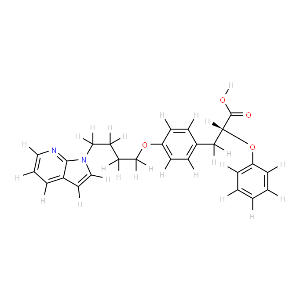 | -0.61 |
| BMCL_06_915_001a | 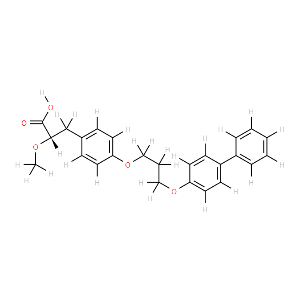 | -0.11 |
| BMCL_06_915_001b |  | -0.57 |
| BMCL_06_915_001c |  | -0.96 |
| BMCL_06_915_001d |  | 0.26 |
| BMCL_06_915_001e |  | -0.25 |
| BMCL_06_915_001f |  | -0.97 |
| BMCL_06_915_001g |  | 0.70 |
| BMCL_06_915_001h |  | 0.67 |
| BMCL_06_915_001i |  | -0.51 |
| BMCL_06_915_001j |  | -0.36 |
| BMCL_06_915_001k |  | -0.74 |
| BMCL_06_915_001l |  | -0.38 |
| BMCL_06_915_001m |  | -0.04 |
| BMCL_99_3329_001 |  | -0.53 |
| BMCL_99_3329_003 |  | 0.83 |
| BMCL_99_3329_004 |  | -0.16 |
| BMCL_99_3329_011 |  | -0.43 |
| BMCL_99_3329_012 |  | 0.79 |
| BMCL_99_3329_013 |  | 1.68 |
| JMC_00_527_GI262570 |  | 3.47 |
| JMC_00_527_GW0207 |  | 1.36 |
| JMC_00_527_GW1929 |  | 2.21 |
| JMC_00_527_GW2433 |  | -0.40 |
| JMC_00_527_GW7845 |  | 3.15 |
| JMC_00_527_GW9578 |  | 0.00 |
| JMC_00_527_L-165041 |  | -0.74 |
| JMC_00_527_L-796449 |  | 2.28 |
| JMC_00_527_SB213068 |  | 1.18 |
| JMC_00_527_Wy-14643 |  | -1.78 |
| JMC_02_789_003a |  | 0.34 |
| JMC_02_789_003b |  | -0.42 |
| JMC_02_789_003c |  | -0.65 |
| JMC_02_789_003d |  | -0.53 |
| JMC_02_789_003e |  | -0.52 |
| JMC_02_789_003f |  | 0.00 |
| JMC_02_789_003g |  | 0.04 |
| JMC_02_789_003h |  | 0.91 |
| JMC_02_789_003i |  | -0.51 |
| JMC_02_789_003j |  | 0.88 |
| JMC_02_789_003k |  | 0.49 |
| JMC_02_789_003l |  | 0.69 |
| JMC_02_789_003m |  | 0.70 |
| JMC_02_789_003n |  | 0.69 |
| JMC_02_789_003o |  | 1.61 |
| JMC_02_789_003p |  | 2.53 |
| JMC_02_789_003q |  | 1.34 |
| JMC_02_789_003r |  | 1.05 |
| JMC_02_789_003s |  | 1.93 |
| JMC_02_789_Benzafibrate |  | -1.30 |
| JMC_02_789_DRF2725 |  | 0.81 |
| JMC_04_196_011 |  | 1.06 |
| JMC_04_196_012 |  | 1.45 |
| JMC_04_196_013 |  | 1.45 |
| JMC_04_196_014 |  | 0.37 |
| JMC_04_196_015 |  | 1.06 |
| JMC_04_196_016 |  | 0.97 |
| JMC_04_196_018 |  | 0.45 |
| JMC_04_196_019 |  | 1.55 |
| JMC_04_196_020 |  | 0.45 |
| JMC_04_196_022 |  | 0.01 |
| JMC_04_196_023 |  | 0.97 |
| JMC_04_196_028 |  | 0.45 |
| JMC_04_196_035 |  | 0.89 |
| JMC_04_196_036 |  | 1.13 |
| JMC_04_196_037 |  | 0.45 |
| JMC_04_196_038 |  | 0.64 |
| JMC_04_196_039 |  | 1.14 |
| JMC_04_196_040 |  | 1.27 |
| JMC_04_196_041 |  | 0.45 |
| JMC_04_196_042 |  | 1.03 |
| JMC_04_196_043 |  | 1.02 |
| JMC_04_196_044 |  | 2.45 |
| JMC_04_196_046 |  | 1.19 |
| JMC_04_196_047 |  | 0.98 |
| JMC_04_196_048 |  | 0.45 |
| JMC_04_196_049 |  | 0.72 |
| JMC_04_196_050 |  | 1.38 |
| JMC_04_196_053 |  | 0.59 |
| JMC_04_196_054 |  | 0.87 |
| JMC_04_196_056 |  | 1.01 |
| JMC_04_196_057 |  | 1.01 |
| JMC_04_3255_003 |  | -0.78 |
| JMC_04_3255_004 |  | -1.16 |
| JMC_04_3255_005 |  | -0.73 |
| JMC_04_3255_006 |  | -0.41 |
| JMC_04_3255_007 |  | -1.20 |
| JMC_04_3255_008 |  | -0.33 |
| JMC_04_3255_009 |  | -0.54 |
| JMC_04_3255_010 |  | -0.89 |
| JMC_04_3255_014 |  | -0.33 |
| JMC_04_3255_015 |  | -0.25 |
| JMC_04_3255_025 |  | -0.21 |
| JMC_04_3255_026 |  | -0.16 |
| JMC_04_3255_027 |  | -0.28 |
| JMC_04_3255_028 |  | -0.78 |
| JMC_04_3255_037 |  | -1.33 |
| JMC_04_3255_038 |  | -0.33 |
| JMC_04_3255_039 |  | 0.46 |
| JMC_04_3255_040 |  | 0.11 |
| JMC_05_5509_008 |  | 0.00 |
| JMC_05_5509_009 |  | -1.35 |
| JMC_05_5509_010 |  | -1.14 |
| JMC_05_5509_011 |  | 0.26 |
| JMC_05_5509_012 |  | -0.60 |
| JMC_05_5509_013 |  | -1.45 |
| JMC_05_5509_014 |  | -0.25 |
| JMC_05_5509_015 |  | -0.22 |
| JMC_05_5509_clofibric_acid |  | -2.53 |
| JMC_98_5020_009 |  | -0.90 |
| JMC_98_5020_015 |  | 0.53 |
| JMC_98_5020_016 |  | 1.62 |
| JMC_98_5020_017 |  | 2.36 |
| JMC_98_5020_018 |  | 2.36 |
| JMC_98_5020_019 |  | 2.90 |
| JMC_98_5020_020 |  | 3.79 |
| JMC_98_5020_021 |  | 1.10 |
| JMC_98_5020_022 |  | 0.18 |
| JMC_98_5020_023 |  | 0.35 |
| JMC_98_5020_024 |  | 0.63 |
| JMC_98_5020_025 |  | 2.62 |
| JMC_98_5020_026 |  | 1.86 |
| JMC_98_5020_027 |  | 0.06 |
| JMC_98_5020_028 |  | 1.29 |
| JMC_98_5020_029 |  | 0.67 |
| JMC_98_5020_030 |  | 0.39 |

**Table S3.** The Best ES descriptors of 178 carboxylic acid PPARγ agonists, directly prioritizing against cellular activity using the Eq 1, without the inclusion of Jurs_RNCG through the Eq 2. The most top ranked ES symbol, ssO, falls outside this monitor table.

| ES descriptors | rank | sign of β*_ES_* |
| --- | --- | --- |
| ES_Sum_aaO  ES_Count_aaO  ES_Sum_dO  ES_Count_dO  ES_Count_sssN  ES_Sum_sssN  ES_Sum_sssCH ES_Count_dssC  ES_Count_ssNH  ES_Sum_ssNH  ES_Count_aaCH ES_Sum_aaaC | 1  2  3  4  5  6  7  8  9  10  11  12 | +  +  +  +  +  +  -  -  +  +  +  + |
